# Empirical Relationship between Intra-Purine and Intra-Pyrimidine Differences in Conserved Gene Sequences

**DOI:** 10.1371/journal.pone.0006829

**Published:** 2009-08-28

**Authors:** Ashesh Nandy

**Affiliations:** School of Environmental Studies, Jadavpur University, Kolkata, West Bengal, India; Aarhus University, Denmark

## Abstract

DNA sequences seen in the normal character-based representation appear to have a formidable mixing of the four nucleotides without any apparent order. Nucleotide frequencies and distributions in the sequences have been studied extensively, since the simple rule given by Chargaff almost a century ago that equates the total number of purines to the pyrimidines in a duplex DNA sequence. While it is difficult to trace any relationship between the bases from studies in the character representation of a DNA sequence, graphical representations may provide a clue. These novel representations of DNA sequences have been useful in providing an overview of base distribution and composition of the sequences and providing insights into many hidden structures. We report here our observation based on a graphical representation that the intra-purine and intra-pyrimidine differences in sequences of conserved genes generally follow a quadratic distribution relationship and show that this may have arisen from mutations in the sequences over evolutionary time scales. From this hitherto undescribed relationship for the gene sequences considered in this report we hypothesize that such relationships may be characteristic of these sequences and therefore could become a barrier to large scale sequence alterations that override such characteristics, perhaps through some monitoring process inbuilt in the DNA sequences. Such relationship also raises the possibility of intron sequences playing an important role in maintaining the characteristics and could be indicative of possible intron-late phenomena.

## Introduction

The apparent lack of pattern of composition and distribution of bases in DNA sequences have been one of the enduring problems of molecular biology. Chargaff's rule provided a clear relationship between the numbers of guanines and cytosines, and between adenines and thymines in duplex DNA sequences, understood well by the Watson-Crick model. However, no clear relationship has been found as yet between the occurrences of these four bases in an individual strand of a gene sequence, although much work have been done on understanding nucleotide frequencies and base distributions in DNA sequences. To cite a few examples, Sueoka [1] proposed a unitary theory based on genetic and evolutionary considerations which attempted to account for the main characteristics of the distribution of DNA base composition in nature. The author considered the GC content of DNA sequences of organisms and mutations of GC base pairs to AT base pairs and vice versa over many generations from which a distribution function of the GC content of DNA molecules at equilibrium was obtained based on assumptions of rather uniform mutation and selection pressures affecting base pair conversions.

Goldman and Yang [2] proposed a codon-based model for the evolution of protein-coding DNA sequences using a Markov process to describe substitutions between codons. They used codon level information to model synonymous and asynonymous nucleotide substitution applicable to homologous sequences with no insertion/deletion gaps or with gaps removed. A 61×61 matrix of codon substitution rates (excluding the three stop codons) is used, assuming that mutations occur at the three codon positions independently and only single-nucleotide substitutions are permitted to occur at any instant. Using several constraints and refinements on the nucleotide substitution rates, the transition/transversion bias and amino acid differences, they show that their codon based model gives better phylogenies than simple nucleotide substitution model. However, the possible patterns arising from this model is very large and computationally very slow requiring Monte Carlo simulations, maximum likelihood methods and other approximations to arrive at quantitative results. While the model is useful for pairwise distance measures and for phylogenies, a relationship defining base composition in a DNA sequence is not clearly realised.

In a recent paper, Qi Ding et al [3] formulated an approach to determine a linear regression model for DNA sequences. By regarding a DNA primary sequence as a random process in time and defining the nucleotides' random distribution functions in three ways based on chemical structures they proposed two methods to measure their similarities. Relating the random distribution functions by a linear regression equation enabled them to construct six new models to analyse the DNA sequences and quantify their similarities and dissimilarities. The optimal model can be chosen based on the amount of information contained or lost in the process.

Several other studies have also focussed on nucleotide asymmetries in DNA sequences to gain an insight into correlations, if any, in base composition and distribution. Arnold et al [4] used tetranucleotide frequencies in a third order Markov chain to predict the frequencies of longer oligonucleotides in the yeast genome and observed that the oligonucleotide frequencies depended strongly on base composition. Freeman [5] considered several prokaryotic DNA genomic sequences and found, from base composition asymmetries like purine excess over pyrimidines and coding strand excess, that the global minima of the purine excesses correlated with the origin of replication, and the maxima with the likely terminus for prokaryotic genomes and that such a prominent correlation between the purine excess and replication direction probably leads to excess pyrimidine accumulation in the sense strand and accordingly should increase the less mutationally vulnerable purine content of the coding strand. Prusak and Grzybowski [6] observed that there is a strong non-random distribution of nucleotides in the cytochrome *b* sequence in several species with the highest differences at the third codon position which is also the codon position of the strongest compositional bias; some species like quail, frog, python and elk appeared to prefer C over A in the light DNA strand whereas species belonging to the artiodactyls contained fewer pyrimidines compared to other species investigated.

Such studies serve to also highlight the complexity of base arrangements in a DNA sequence and the difficulties in finding any inherent pattern or signal sequences in such arrangements, especially in a character based representation of the hundreds and thousands of nucleotides comprising the sequences. However, a graphical representation can be expected to provide visual clues to any inherent pattern or regularity as distinct from a purely random distribution of the bases which could be expected to generate a corresponding random distribution in the representative plot. Indeed, a purine-pyrimidine sequence map proposed by Peng et al [7] provided a visual rendition of the growth of purine and pyrimidine numbers in a DNA sequence, which was interpreted by the authors as indicative of an inherent fractal nature in the purine-pyrimidine structure of the DNA sequences. The chaos generator representation of Jeffreys [8] with its double-scoop depletion pattern reflected largely the abundance, or sparseness, of various dinucleotides and higher combinations, and also showed characteristic variations for different classes of organisms.

Graphical representations of DNA sequences assigning the four individual nucleotides to designated axes on a Cartesian grid provide a more direct visual indication of the progression of nucleotides in a sequence, akin, in some respects, to the Wilson cloud chamber for particle tracks. The first graphical representation of a DNA sequence was proposed by Hamori and Ruskin [9] based on a 3D Cartesian axis system that generated a visual map of the sequence of bases in a selected DNA sequence. A 2D representation was proposed by Gates [10] and rediscovered independently by Nandy [11] and Leong & Morgenthaler [12], with different axes identifications for the four bases. These were followed subsequently by many different approaches to render a visual representation of the DNA sequences [13], [14], and their applications have been found to be very useful in elucidating different characteristics of DNA sequences that are not easily accessible in other ways. For example, Gates [10] referred to large scale structures seen in the plots of some sequences; such structures were also reported by Nandy and Nandy [15], and recently by Larionov et al [16] who reported, inter alia, the presence of gigantic palindromes in mouse and human chromosomal sequences. Liao et al [17] have shown that 2D graphical methods that convert a DNA sequence to a series of co-ordinates and therefrom construct distance matrices can be used for computation of molecular phylogeny without need for multiple alignments; Wang et al [18] constructed a 3D representation in which a DNA sequence could be denoted mathematically and a similarity matrix constructed for multiple sequences to derive a phylogenetic tree by virtue of the fuzzy theory. Lo et al [19] have shown using a 3D trajectory method that global views of DNA sequences can be obtained such that different types of DNA sequences can be easily distinguished and any local differences and similarities between two DNA sequences can also be easily observed.

Furthermore, numerical characterisation techniques based on graphical representations have enabled quantitative estimations of sequence similarities and dissimilarities [see review 14]. Basically there have been two approaches for numerical characterization, both of which use the graphical representation to map a DNA sequence into a set of numbers. One approach using geometrical mapping proposed by Raychaudhury and Nandy [20] have been found to be useful for several calculations based on the 2D graphical representation [14], and extended recently to an abstract 20D modelling for protein sequences [21], where individual sequences are indexed by numerical descriptors. The other approach is to use matrix methods by forming ratios of graph theoretic and Euclidean distances between nodes of the graphical plots, first formulated for DNA sequences in Randic et al [22]. Since invariants associated with matrix formulation are well-known, individual sequences can be indexed by one or more such invariants of various orders; it is expected that these would be sufficiently characteristic of the underlying sequences to enable unique characterization. This technique has been the most widely used method of choice for the researchers in this field who have defined different types of matrices to construct various invariants to describe the DNA sequences. However, the difficulties associated with computing various parameters for very large matrices that are natural for large sequences have restricted the numerical characterizations to leading eigenvalues and the like [14].

In principle, however, many of the indices used to characterize numerically DNA representations are graph invariants that describe the distribution of nodes and/or node-node connections in these graphs. In the parlance of graph theory, many authors have referred to some of these indices as Topological Indices (TIs) and applications have been made not only to DNA sequences but also to proteins, viral surfaces, RNA secondary structures and small molecules [23], [24]. Consequently, the method is of more general application taking into consideration that the type of graph representations referred to above have been extended from DNA/RNA to the study of other types of relevant biological sequences. In particular, González-Díaz et al. extended these representations to the study of protein sequences [25] and Mass Spectra outcomes of proteins and/or protein serum profiles in parasites [26], toxicoproteomics and diagnosis of cancer patients [27], [28]. In any case, the various numerical parameters of DNA/RNA graph representations (TIs or otherwise) may be used not only to study sequence-sequence similarity but also to fit Quantitative Structure-Activity Relationship (QSAR) models. These QSAR connect structural information with the biological function of a molecular entity under study and may be used to predict unknown entries. Structure here refers not only to drug structure but also to DNA sequence, RNA sequence or secondary structure, and protein sequences or 3D structure [see review 28].

Thus, the utility of graphical methods in revealing different types of hidden structures and similarities/dissimilarities in and between DNA and other biological sequences can be considered to be well demonstrated.

In this light, a perusal of the representative patterns of conserved gene sequences appears to indicate a possible relationship between the numbers of the various nucleotides in conserved gene sequences. Here we use the 2D graphical representation method to show that plots of the conserved gene sequences trace out apparently curved paths that are also visually similar across species for the same gene. The nature of these curves is seen to generally imply a so far undescribed quadratic relationship between intra-purine and intra-pyrimidine differences, whereas the null hypothesis would have indicated random directionless walks. With such empirical relationship between the two basic nucleotide differences, we propose a probable mutation path to explaining the relationships. We then hypothesize that the parameters of such relationships could be a property of the underlying gene sequences and speculate that extensive alterations of such genes by accretion or deletion of DNA fragments would be s only if the modified sequences subscribe to the same basic parameterised relation.

## Methods

**Figure 1:**
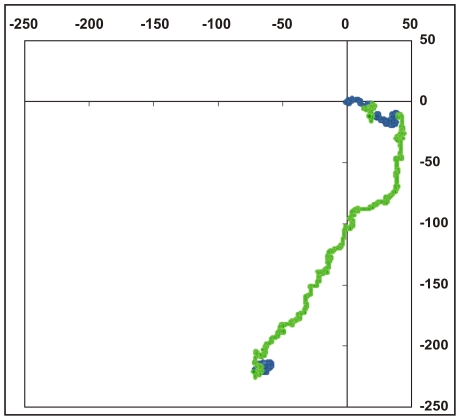
2D graphical representation of the human beta globin gene, complete cds from HUMHBB (GenBank). Intron regions are coloured green, exons blue. The axes are A, C, G, T clockwise along the four cardinal directions starting from the negative x-direction as explained in the Methods section.

Here we use the random walk system envisaged in the Nandy plot [11] based on a 2D Cartesian grid where the four bases are assigned to the four cardinal directions: guanine (g) to the positive x-direction, thymine (t) to the negative y-direction, adenine (a) to the negative x-direction and cytosine (c) to the positive y-direction. The method to plot a DNA sequence is to start at the origin and take a step in the positive x-direction for a guanine base, in the negative x-direction for an adenine, positive y-direction for a cytosine and the negative y-direction for a thymine, and proceed likewise for each succeeding base in the sequence, starting each step from the end of the last one taken. This way a succession of bases in the original DNA sequences is represented by a succession of points in the 2D plot, the overall trace being a representation of the distribution of bases in the DNA sequence (see e.g., Fig. 1 – human beta globin). The axes essentially represent the excess of guanine over adenine along the x-axis and the excess of cytosine over thymine along the y-axis; thus the plots are basically of instantaneous values of intra-pyrimidine, intra-purine differences as we proceed along the sequence. The end point of such a curve will be given by (*N_G_-N_A_, N_C_-N_T_*), where by N_A_, N_C_, N_G,_ N_T_ we mean the total number of adenines, cytosines, guanines and thymines in the sequence being plotted. GC-rich sequences therefore plot mostly in the first quadrant, AT-rich sequences in the third quadrant on this axes system.

Once the co-ordinates are available, we use an Excel spreadsheet to plot the graph and apply the *Add Trendline* feature of the Excel software to fit the best polynomials for our analysis, with axes transformation where required to conform to the software's curve fitting engine.

## Results

**Figure 2:**
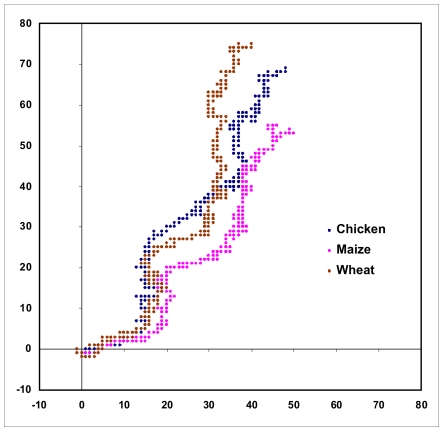
2D graphical representations of three histone H4 sequences: Wheat (brown), maize (pink) and chicken (blue). The three plots can be seen to have similarities in shape.

**Figure 3:**
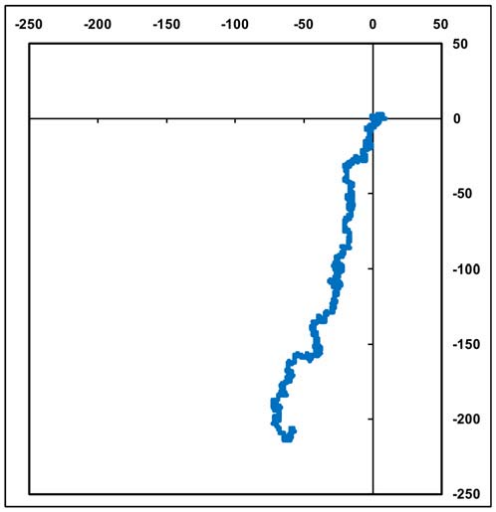
The 2D graphical plot of the randomised human beta globin gene. This is done as per the on-line randomisation tool in Ref.[14].

Fig. 1 shows a plot of the human beta globin gene complete cds generated using the above algorithm. We note that a DNA sequence that consists of a succession of short segments each having a complete mix of a,g,c,t with equal contributions of each of the bases within each of the segments would be expected to generate a dense cluster of points around the origin; a random distribution of the a,g,c,t along the sequence could be expected to generate a random walk. The human beta globin gene sequence complete cds inclusive of all introns and exons (Fig. 1) shows a distinct pattern where the bases appear to follow one another with some regularity, with the total extent of the representative plot arising from the non-equal composition of the bases in the sequence; other beta globin sequences produce similar plots implying that the human beta globin gene cds is not an arbitrary random sequence. Fig. 2 shows the close similarity of the shapes of the plots of three sequences of histone H4 genes of wheat, maize and chicken, demonstrating that these sequences are not random but have a close kinship in base distribution. A randomisation of the bases in the human beta globin gene [29] produces, on the other hand, a simple linear plot (Fig. 3) in the third quadrant of the axes system as can be intuitively expected for an unorganised mixture of the four bases along the sequence.

**Figure 4:**
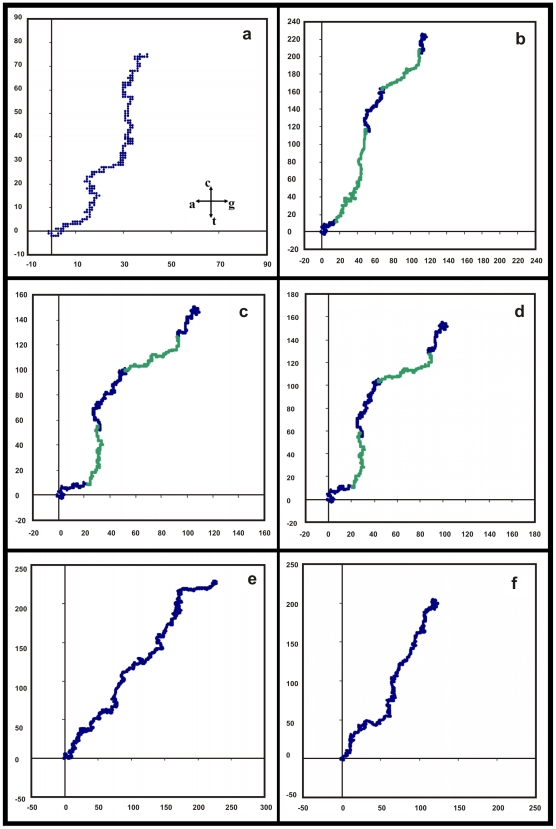
Plots of six selected conserved gene sequences in a 2D graphical representation. Axes representation as in Fig. 1 and also inset in Fig. 4a. Intron segments are shown in green, exons in blue. (a)Wheat histone H4 TH091 gene (GenBank Locus ID WHTH4091) (b) Horse BI alpha-1 globin gene (HRSHBA22) (c) Rhesus monkey alpha-globin gene (MCHBA) (d) Human hemoglobin alpha 1 (HBA1) gene (from HUMHBA4) (e) Trypanosoma cruzi hsp70 gene (HSHSP70) (f) Chicken beta-1 tubulin gene (CHKTUBB1)

**Figure 5:**
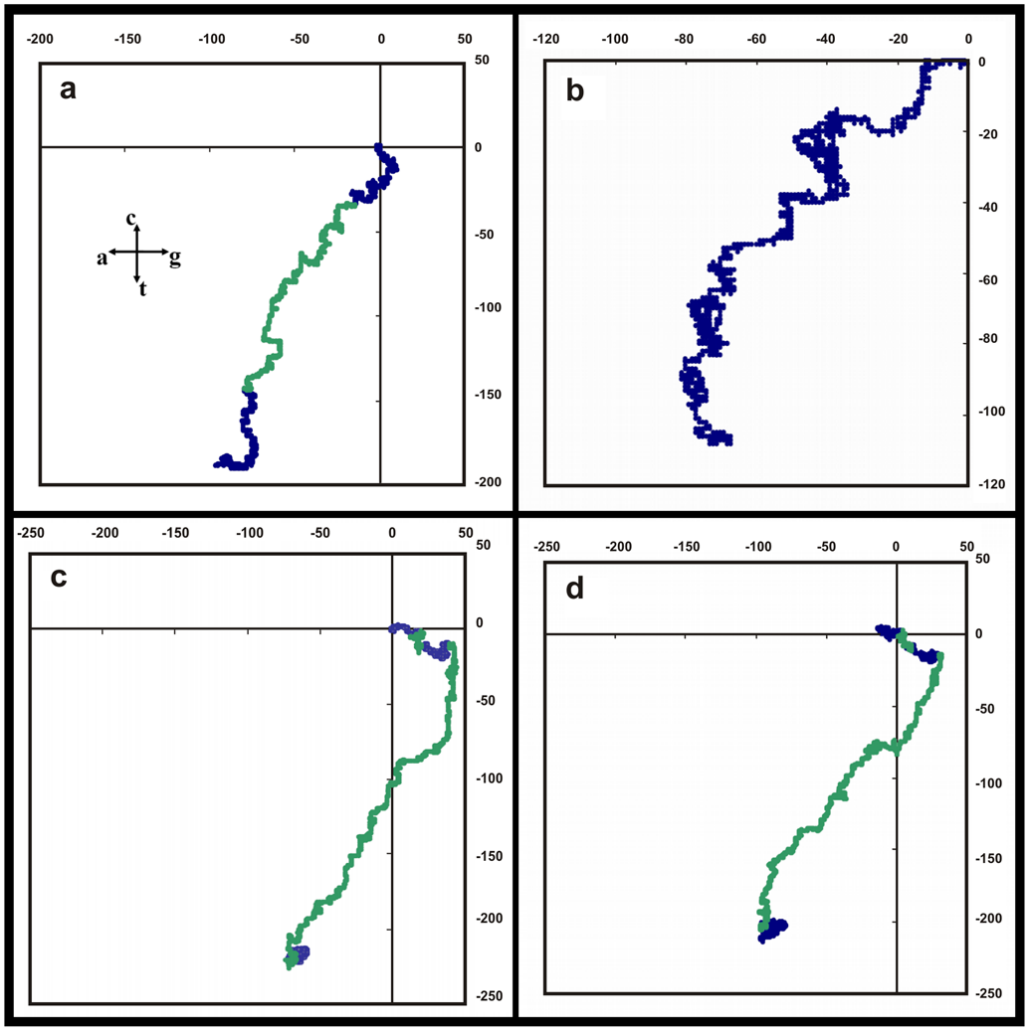
Plots of four more sequences in continuation of Fig. 4. (a) Soybean heat-shock protein (Gmhsp26-A) gene (SOYHSP) (b) H5N1 neuraminidase of A/Indonesia/CDC10327/2007 (CY019402) (c) Human beta globin gene (from HUMHBB) (d) Human delta globin gene (from HUMHBB).

To further demonstrate that the base distribution in these gene sequences are non-random, and generally true for conserved gene sequences, we have generated graphical representations of several conserved genes, a selection of which are shown in Fig. 4. Plots of several alpha globin genes are included here to show that there is shape similarity between the same genes from different species indicating that entire gene sequences inclusive of introns and exons have close similarities. This can also be seen in the plots of several histones, tubulins and heat shock proteins, of which some representative samples are given in Figs. 4 and 5.

**Figure 6:**
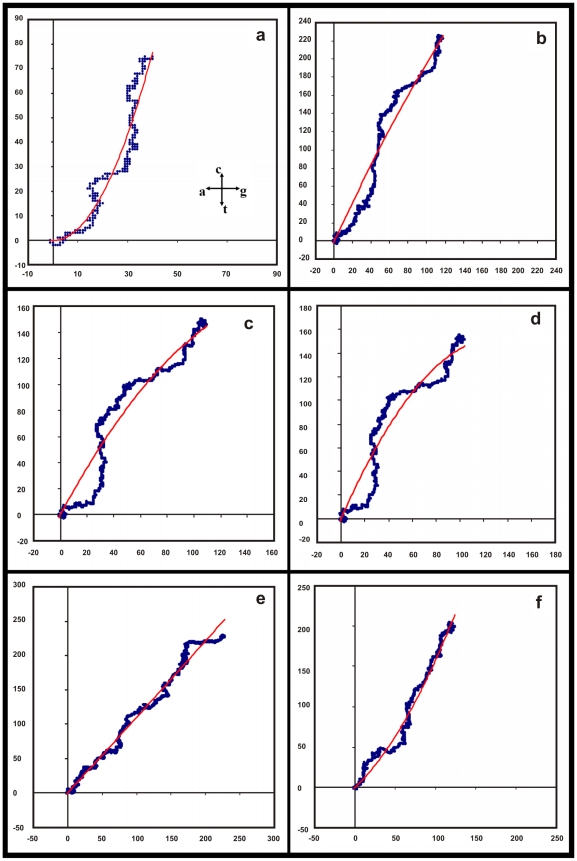
Plots of the six gene sequences in Fig. 4 with quadratic polynomial fits. Labels for (a) to (j) are given under Fig. 4.

**Figure 7:**
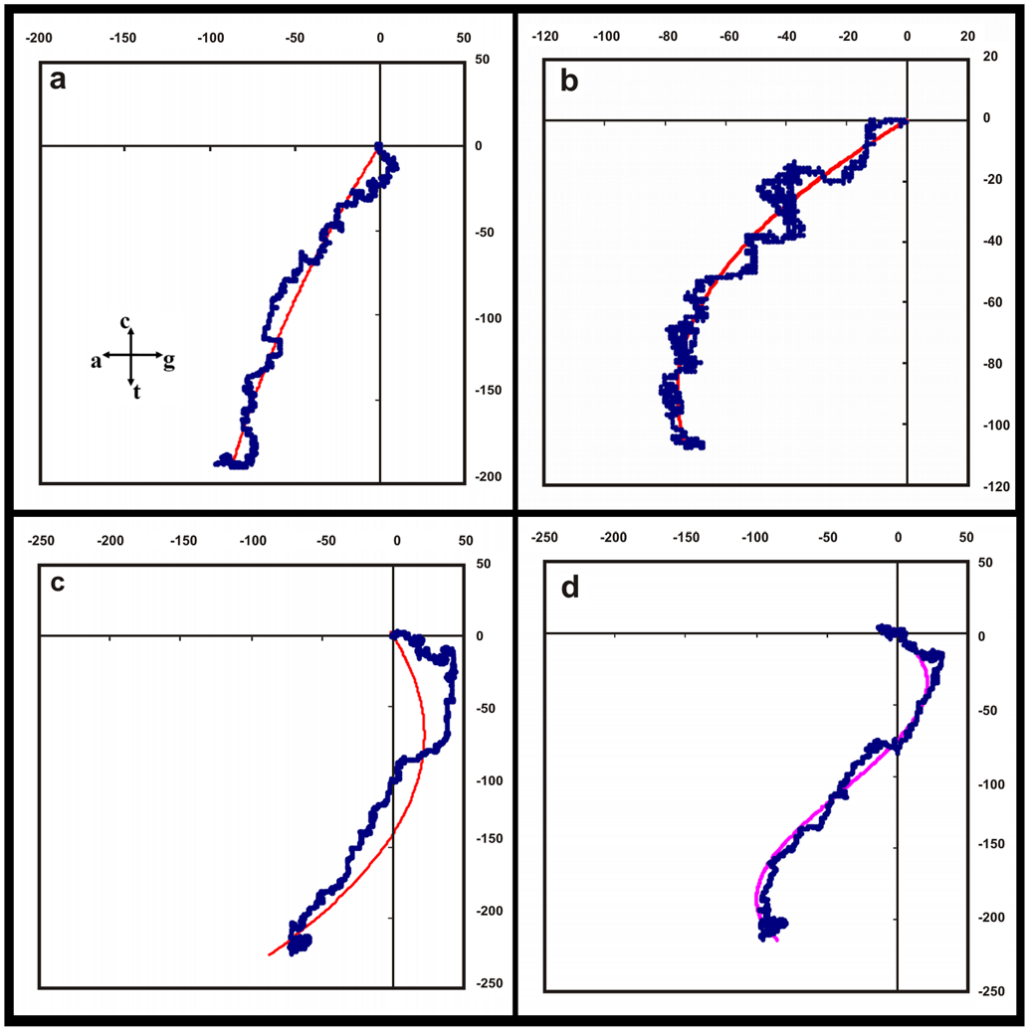
Plots of the four sequences of Fig. 5 with polynomial fits. All are quadratic fits except for the human delta globin gene where the cubic fit is shown.

With our observation that the sequences of the conserved genes, both intronless and with-introns, have close similarities, we can start to enquire whether these sequences have any discernible patterns. We notice that the general nature of the DNA walks on the 2D representation as per the Nandy prescription [11] shown in the above figures is directional and curvilinear. A simple 2^nd^ degree polynomial produces reasonable fits. A selection of the plots with the trendlines is shown in Fig. 6 and 7 corresponding to the sequences shown in the previous two figures. A list of the details of the fits and statistics are given in Table 1.

**Table 1:** Gene sequences and coefficients of polynomial fits.

Gene Sequences, complete cds	GENBANK Locus	Sequence type	Polynomial Coefficients			*R^2^*
			Linear	Quadratic	Cubic	
				*Quadratic Fits*		
*Trypanosoma cruzi* hsp70	HSHSP70	GC-rich	1.0934	7.00E-05		0.9809
Petunia hsp70G	PHHSP70G	AT-rich	0.2847	4.00E-06		0.846
*C elegans* hsp16C	CEHSP16C	AT-rich	1.1824	0.0002		0.9848
*D pseudobscura* hsp82	DPHSP82	AT-rich	1.4998	0.0041		0.9097
Soyabean hsp 26-A	SOYHSP	AT-rich	0.6283	0.0009		0.9473
Human alpha globin 1	HUMHBA4	GC-rich	2.2858	−0.0085		0.8912
Horse alpha globin	HRSHBA22	GC-rich	2.1736	−0.0022		0.9316
Rhesus monkey alpha globin	MCHBA	GC-rich	1.9069	−0.0053		0.9044
Goat adult alpha-globin	GOTHBAI	GC-rich	0.5345	0.0087		0.9705
Chicken alpha globin	V00410	GC-rich	4.8816	−0.0456		0.7963
Human alpha 1 pseudogene	HUMHBA4 (HBAP1)	GC-rich	3.4216	−0.0487		0.538
Chicken beta-1 tubulin gene	CHKTUBB1	GC-rich	0.9464	0.0063		0.9761
Wheat histone H4	WHTH4091	GC-rich	−0.0071	0.0481		0.8479
Maize histone H4C13	MZEH4A	GC-rich	0.9235	-		0.8235
Chicken histone H4	CHKHIST4A	GC-rich	0.7713	0.0153		0.9383
Human beta globin	HUMHBB	AT-rich	−0.631	-0.0045		0.8073
Goat alanine beta globin	GOTHBBAA	AT-rich	−1.4243	−0.0121		0.6786
Mouse beta-1 globin	V00722	AT-rich	−1.2538	−0.0093		0.0612
Chicken beta globin	V00409	GC-rich	0.9817	−0.0049		0.7268
Oppossum beta globin beta-M	OPOHBBB	AT-rich	0.5623	5.00E-05		0.9431
*X tropicalis* larval beta globin	Y00501	AT-rich	1.5184	0.0013		0.9493
*H5N1 neuraminidase genes:*						
Duck/Guangdong/07/2000	AY585404	AT-rich	1.4465	0.0075		0.7659
Duck/China/E319-2/03	AY518363	AT-rich	1.2828	0.0071		0.8776
Bar-headed goose/Qinghai/0510/05	DQ137874	AT-rich	1.3274	0.0081		0.8022
Indonesia/CDC10327/2007	CY019402	AT-rich	1.6888	0.0094		0.9126
Peregrine falcon/Hongkong/2142/2008	CY036271	AT-rich	1.2120	0.0060		0.8586
				*Cubic Fits*		
Mouse beta-1 globin	V00702	AT-rich	−2.6112	0.0404	−0.0002	0.5286
Human beta globin	HUMHBB	AT-rich	−1.7856	0.0214	−5.00E-05	0.94
Human delta globin	HUMHBB	AT-rich	−1.3405	0.0231	−7.00E-05	0.9757

While this nature of the base distributions is found across many different sequences of eukaryotic genes, it is also very much evident in the case of viral sequences like the H5N1 neuraminidase which are known to mutate very rapidly; other plots from a sample of over 600 sequences of the H5N1 neuraminidase show similar quadratic forms. Interestingly, plots of the wheat, maize and chicken histone H4 genes, which are also intronless genes, can also be fitted by polynomials of degree 2, similar to the case of the viral genes. Chicken beta globin gene with intron sequences that are quite different compared to the mammalian genes plot in the first quadrant, but it also can be fit by a quadratic polynomial.

This is however not true of all gene sequences. The mouse beta globin gene sequence representation in the 2D framework gives a very poor fit for the quadratic function (*R^2^ = 0.06*) but much better statistics with a cubic polynomial (*R^2^ = 0.53*) (Table 1); the human delta globin gene also shows very good statistics when fitted with a cubic polynomial (*R^2^ = 0.98*). Some sequences where significantly large segments are at variance with the overall pattern too cannot be put into such slots. The rat myosin heavy chain gene sequence where the intron sequences have been hypothesized to have grown through accretion of large fragments [30], for example, presents a highly compact form on the 2D plot [15] and cannot be fit by the simple polynomials we have used so far. However, sufficiently large numbers of sequences are seen to follow the apparent quadratic relationship with good statistics that it is of interest to try to understand the underlying pattern.

## Discussion

The equations that fit the curvilinear patterns of the base distributions with reasonable statistics are of the form
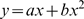
(1)and
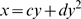
(2)where a,b,c,d are parameters,
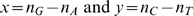
(3)and n_A_, n_C_, n_G_, n_T_ are the instantaneous values of the numbers of a, c, g, t present up to the particular position (*x,y*) on the sequence, starting the count from the beginning, i.e. the 5′-end, of the sequence. These are our empirical equations connecting the intra-purine (*x*) and intra-pyrimidine (*y*) numbers obtained from the observations of the patterns on the 2D graphical plots. While the majority of the plots shown are well-represented by such polynomials of the second degree, the fits could be improved in some instances by fitting higher degree polynomials as mentioned earlier; e.g., in the case of the human beta-globin gene a polynomial of the third degree yields better statistics (*R^2^ = 0.94*) than the second degree (*R^2^ = 0.81*), for the human delta globin gene the statistics for the quadratic and cubic fits are *R^2^ = 0.87* and *R^2^ = 0.98*, respectively. We, however, consider the second degree form for now for conformity without excessive loss of statistical significance.

The origin of such a relationship as in equations 1 and 2 could be traced to mutational changes in a sequence, where we restrict our analysis for the moment to transitional types of mutations since this is the dominant mode. Consider a mutation of a cytosine to thymine in one strand of a DNA in a GC-rich sequence. The opposite strand, calling it strand 2 for convenience, will initially have a bulge for the original paired guanine, and the event leads to following possibilities [31]: (a) the DNA repair mechanism reverse mutates the thymine to cytosine in strand 1, thus negating the effect of the original mutation; (b) the guanine in strand 2 is replaced by an adenine; and (c) a third possibility in case the damage repair coincides with replication, that the DNA is elongated by pairing the mutated thymine in strand 1 with a new adenine in strand 2 and addition of a cytosine in strand 1 to match the guanine in strand 2 left over after the original mutation, i.e. creation of a T-A pair and addition of a new C-G pair. Such an event would be quite rare, especially in coding regions since it will alter the reading frames unless the total change leads to addition of three base pairs; several intronless gene sequences indeed show very small contribution from the quadratic term compared to the interrupted genes, e.g. petunia hsp70G (Table 1). The example of the mutation event of cytosine to thymine in strand 1 can be considered to change the intra-pyrimidine difference, n_C-T_, in strand 1 and trigger a change in the intra-purine difference, n_G-A_, in strand 2. The two changes can be related by
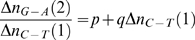
(4)where the first term on the right relates to the probability of the G to A change in strand 2 and the second term to the probability of the third type of response, i.e. DNA elongation by addition of a C in strand 1; the (1) and (2) in equation 4 are strand identifiers and we have defined
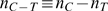
(5)

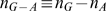
(6)


We expect q<p since the probability of DNA elongation will be significantly lower than effecting only a replacement of the guanine of strand 2 by an adenine. We then have
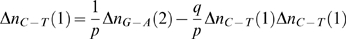
(7)


Using recursion and keeping up to first order terms in q/p, equation (7) reduces to
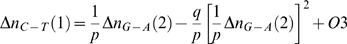
(8)where by *O3* we mean terms of higher orders (dropping the conventional form *O(3)* so as not to confuse with the strand number indicators). For many mutations over a long sequence, this takes the form
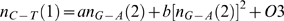
(9)where a,b are redefined constants with b<a. This equation relates changes in the intra-pyrimidine numbers in strand 1 to intra-purine numbers in strand 2 where the n_C-T_ and n_G-A_ are as defined in equations 5 and 6 above.

Now, Chargaff's rule states that
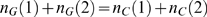
(10)

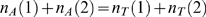
(11)and since from Watson-Crick rule we know 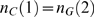 and 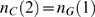, and similarly for A,T, then from the above we have
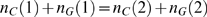
(12)

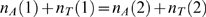
(13)as a consequence of which, 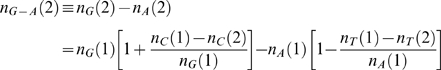



Excluding some pathological cases or very short segments where one or the other type of nucleotide is conspicuous by its absence, e.g., as in poly-adenylation segments where only A's dominate and the others are absent, in a real gene sequence we can expect the second term within each of the square brackets above to be≪1, implying that
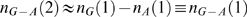
(14)which transforms Eq. (9) to
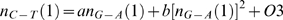
(15)or, dropping the strand number indicator since all quantities now refer to the same strand,
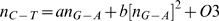
(16)


From similar considerations for the AT-rich sequences considering the mutations of adenine to guanine for example, we can obtain the equation
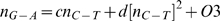
(17)where *n_C-T_* and *n_G-A_* are as defined in Eqs. 5 and 6. Equations 16 and 17 are similar to equations 1 and 2 and conform to the general shapes of the sequence plots. This shows that the path traced out by the nucleotide sequence of a gene follows some pattern that can be ascribed to the accumulated effects of spot mutations over evolutionary time scales. The actual plots of the gene sequences in the 2D grid representation show reasonable conformity with the predicted curves with variations that could be ascribed in part to the higher order terms and other factors described below. It is to be noted that equations 16, 17 have been derived independent of any graphical representations or reference frames, and they are functions of the instantaneous values of the base counts only. The 2D Nandy representation here used happens to be a natural and convenient reference frame to plot the outcome of these equations.

Equations 16–17 have been developed on the basis of transition type mutations only. Transverse mutations are much less frequent than the transition mutations and will affect these equations to a smaller extent. Consider a mutation of a cytosine to an adenine. This will result again in a bulge in the guanine in the opposite strand and the repair mechanism will either re-establish the cytosine or replace the guanine with a thymine, or replace the guanine in strand 2 with a thymine and add a CG base pair to the DNA thus elongating it. This will reduce the *n_C-T_(1)* by 1 unit and also reduce the *n_G-A_(2)* by 1 unit as well as have, as before, a small probability of adding an extra guanine in strand 2 by virtue of the additional base pair elongating the DNA. Thus we will have an equation of the type of Eq.4 again except that the coefficients in this instance will be much lower in value than for these parameters in the transition type mutation case. Again, we end up with equations of the form 16 to 17, this time for transverse type mutations, but with much smaller parameter values, so those equations can be considered to be quite general.

It is also possible that as a result of mutations more than one base pair are added during the repair and replication phases. Such actions can be expected to lead to further higher order terms being added to equations 16, 17. That the third order terms seem to be required for good fits to some of the gene sequences has already been noted earlier. Such modifications to the intra-purine, intra-pyrimidine relationship can result in deviations from the smooth distribution that first order approximation equations like Eq.16, 17 would imply. This could be the underlying reason for the deviations between the actual representative plots of the various sequences and their fitted curves observed in several graphs.

There is a further assumption that underlies the applicability of this analysis. We assume that in the case of the conserved gene sequences inclusive of introns and the coding segments, extensive restructuring through recombinations have not taken place. Indeed the first order formalism developed here implicitly assumes that all changes to a DNA sequence that have resulted in the form that we observe now have taken place through mutational changes only, and recombinations and transpositions have not had any major impact on the sequences.

**Figure 8:**
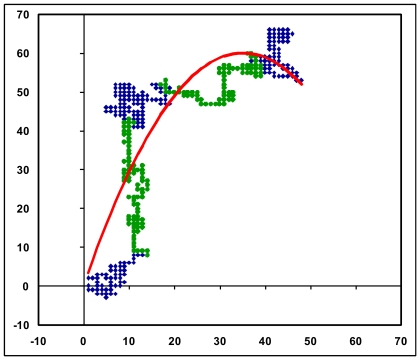
Plot of the human alpha globin 1 pseudogene. The plot shows the introns and exons and the quadratic fitted curve in separate colours.

A couple of points of interest arising from these equations may be mentioned here. First, homologous sequences of conserved genes of the intronless or with-introns varieties that have the same general shape on the 2D plots therefore have similar describing equations, and the coefficients of the variables have similar order of magnitude values. This would seem to indicate that these gene sequences have inherent characteristics that are expressed by the values of the parameters of the describing equations, whereby major deviations in base distributions that necessitate large departures from the characteristic values could be inimical to the functioning of the gene and thus would either be rejected, or would render the gene ineffective. A case could be made from the human alpha globin 1 pseudogene: although it shares a reasonable degree of homology with the functional alpha 1 globin gene and has the 3 exon-2 intron architecture of the globin family, its 2D plot shows a wide variation in base distribution from the alpha globin gene (Fig. 8), and the coefficients of fitted polynomial also are quite different from the other mammalian alpha globin family fits (Table 1). It may also be mentioned that transposons are found in many instances to insert segments into genes which are then excised out in successive replication cycles. If DNA sequences have inherent characteristics, which are encapsulated by the polynomial expression, and the inserted segments lead to incompatibility with such characteristics, then such excisions can be understood.

We note that reversion of mutated genes to ancestral forms is not totally unknown. A case in point is the recent study of reversion of mutant hothead gene in *Arabidopsis thaliana* to genes that existed in plants of two or more generations ago [32]. The authors have hypothesized that a template-directed restoration of ancestral DNA passed on in an RNA cache could underlie the mechanism of such reversion; the existence of such a mechanism that lies outside the DNA genome could lead to the high-frequency modification of DNA sequences in a template-directed manner, perhaps by the postulated RNA cache that could allow it to persist for several generations. While Lolle et al's RNA cache [32] would need to carry an exact duplicate of the ancestral sequence, in the case of gene sequences of the types considered here that may or may not contain intron segments and could be quite large, we could postulate existence of some as yet unknown mechanism for monitoring conformity with the overall intra-purine intra-pyrimidine base distribution pattern, as perhaps for long range correlations [7], [33]. In this connection it is also interesting to consider the possibility of the existence of some error correcting code in DNA sequences as speculated upon by Liebovitch et al [34]. They considered the DNA sequence as a digital code of four symbols and speculated that since the integrity of modern information encoding is secured by having error correcting codes built in, DNA sequences might also have such codes to allow repair enzymes to protect the fidelity of nonreplicating DNA and increase the accuracy of replication. In such a case if a linear block error correcting code is present in DNA then some bases would be a linear function of the other bases in each set of bases. Although the authors were unable to find any such simple code in the lac operon and cytochrome c gene they investigated, the suggestion remains an intriguing possibility nevertheless. Given that we are considering highly conserved genes whose functions are important to the survival of the organism, mechanisms such as these would provide a survival advantage and could be used under conditions that compromised the continued functioning of the organism or the requirements of the monitoring process.

Second, the basic patterns, and therefore the describing equations, have the same form irrespective of the intron content of the genes considered here. This could be indicative of the monitoring process hypothesized above also functioning irrespective of whether the sequences are intronless or intron-rich. Since mutations and other evolutionary changes have led to modifications in the coding sequences within the requirements of maintaining protein functionality, the introns may have a role to play such as maintaining the structure of base arrangements so that the restrictions implied by the equations 16, 17 can continue to apply. We could perhaps consider for support for this contention the globin genes where the beta globins separated from the alphas quite late, but the introns of the beta globin cluster are generally longer than the alpha globins while the coding sequences, though with differences, remain at almost the same length. If sustained, such a hypothesis could lend support to the intron late theory.

We note in passing that for the majority of the gene sequences of the vertebrates, the 2D plots display an overall shape that is concave going in the clockwise direction; correspondingly, the coefficient of the second degree variable is negative. This is seen even in the case of the chicken beta globin gene where the large intron component makes it GC-rich, the human beta globin gene which is AT-rich, as also the alpha globin genes of the horse, rhesus monkey and human which are all GC-rich. Plots of some viral sequences such as the H5N1 neuraminidase, on the other hand, have concavity in the opposite direction and the sign of the coefficient of the second degree variable is positive. Interestingly this is also seen in the case of the wheat histone H4 gene, which is claimed by some authors to have viral features [35]; their origins from before the eukaryote split could also be a factor. Whether this is just chance coincidence or whether it is symptomatic of some deeper characteristic arising from base composition and distribution differences in prokaryotic and eukaryotic sequences remains an intriguing question.

In summary, our observations have shown that the 2D plots of intra-purine versus intra-pyrimidine differences in conserved gene sequences exhibit an apparent pattern in base distribution of the sequences that mimic the behaviour essentially of a polynomial of degree 2, and in some cases of degree 3. This is found over a wide cross section of sequences, from e.g., the members of the globin family, the histones, tubulins and heat shock proteins. Viral sequences such as the H5N1 neuraminidase, although known to mutate rapidly, also exhibit similar structure. We have seen that this may arise from the non-symmetrical mutation repair mechanism where e.g., a cytosine mutating to thymine in a GC-rich sequence could lead to negating the mutation, to replacing the original paired guanine with adenine, or elongating the DNA by addition of a CG pair along with coupling the thymine with an adenine. Equivalent considerations apply to AT-rich sequences as well.

Since these observations appear true for intron-rich sequences also, the intron sequences may play a regulatory role in preserving sequence integrity as indicated by the intra-purine intra-pyrimidine relationships permitting greater flexibility in changes in coding sequences.

Not unexpectedly, we have seen that homologous genes have characteristic equations where the coefficients of the describing polynomials are quite close. Assuming that the DNA fidelity processes fit to this scheme of preferential arrangement of bases in conserved segments, our observations raise the possibility that DNA fragments, introduced into such segments by processes such as transpositions, that do not conform to the overall fit may be preferentially excised by the replication machinery to retain the integrity of the host sequence. If our observations here in gene sequences are extendible further to genomic sequences then it would imply that not all genetic modifications would be sustainable.
